# Supplementary material for: Zwitterions Layer at but Do Not Screen Electrified Interfaces
Source: J Phys Chem B. 2022 Feb 23;126(8):1852–60. doi: 10.1021/acs.jpcb.1c10388 (PMC8900129; doi:10.1021/acs.jpcb.1c10388)
Supplement: Supplementary file 1 — jp1c10388_si_001.pdf [file jp1c10388_si_001.pdf]

Supporting Information

Zwitterions Layer at but Do Not Screen Electrified Interfaces

Muhammad Ghifari Ridwan, Buddha Ratna Shrestha, Nischal Maharjan, Himanshu Mishra*

Environmental Science and Engineering (EnSE) Program, Biological and Environmental Science and Engineering (BESE) Division, King Abdullah University of Science and Technology (KAUST), Thuwal 23955-6900, Saudi Arabia

Interfacial Lab (iLab), Water Desalination and Reuse Center (WDRC), King Abdullah University of Science and Technology (KAUST), Thuwal 23955-6900, Saudi Arabia

*Corresponding Author: himanshu.mishra@kaust.edu.sa

Force-distance curves for the silica–silica system obtained via colloidal probe AFM with probe-tips of radius 6.6 µm.

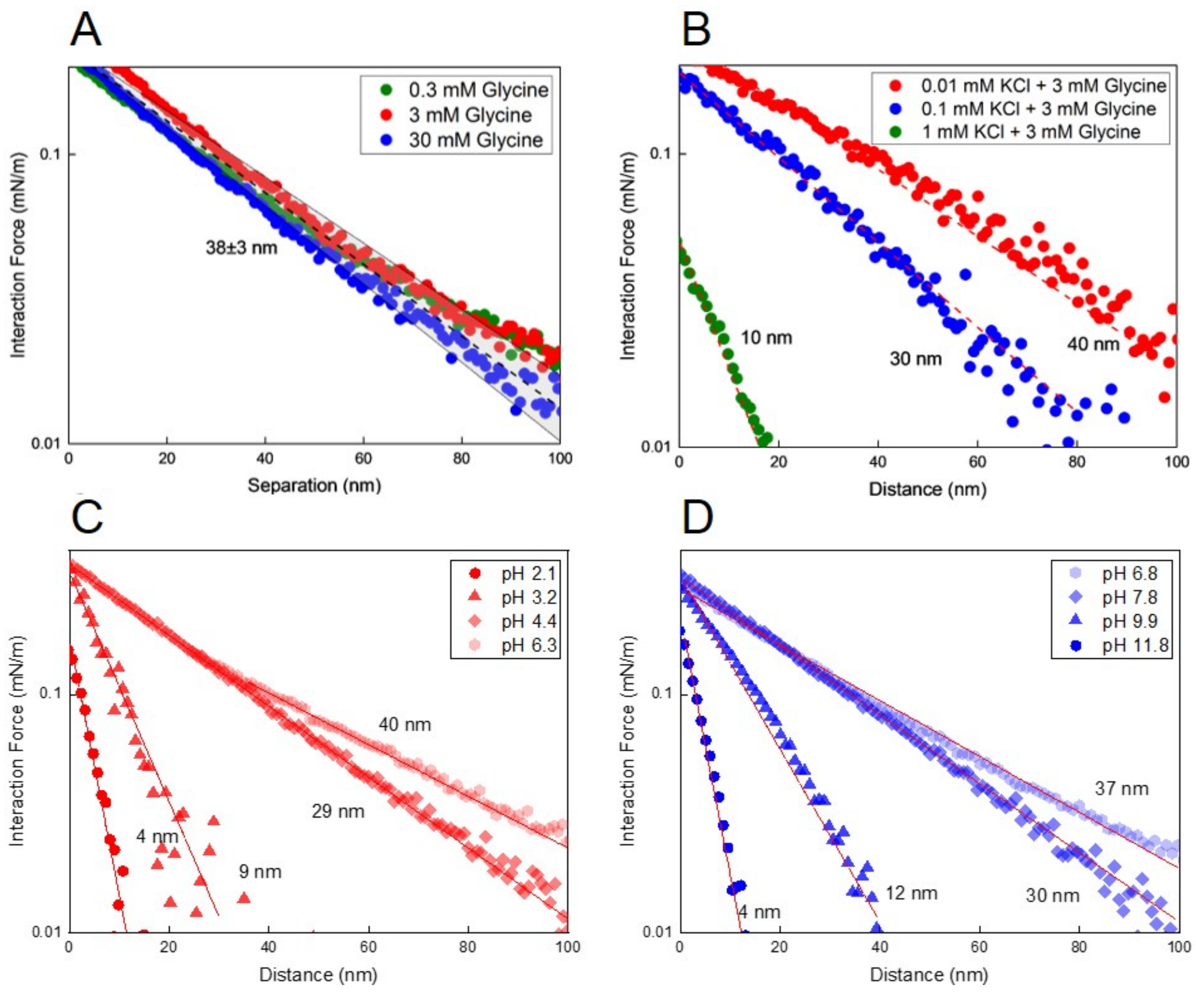

Figure S1. Effects of glycine on the screening lengths and the normalized force–distance curves for the silica–silica system probed via AFM using colloidal probes of radius 6.6 µm. (A) Effects of glycine concentration in the range 0.3–30 mM on the measured interaction forces. Dashed black lines are the best fits obtained using the equation $\frac{F}{R} = \left\{\frac{64\pi\varepsilon_o\varepsilon_r}{\lambda}\left(\frac{k_B T}{e}\right)^2 \tanh^2\left(\frac{e\psi_o}{4kT}\right)\right\} e^{-\frac{D}{\lambda}}$, where $\varepsilon_o\varepsilon_r$ is the permittivity of the medium, e is the electronic charge, k_B is the Boltzmann constant, and T is the absolute temperature, I is the ionic strength, λ is the Debye length, D is the separation distance between two surfaces and ψ_o is the surface potential[1]. The shaded area represents the errors associated with screening length estimation (λ). (B) Effects of adding KCl to glycine solutions on the normalized force–distance curves for the silica–silica system probed via colloidal probe AFM. Normalized force as a function of distance between interacting surfaces 3 mM glycine solutions under (C) acidic and (D) basic conditions.

Speciation of glycine with pH.

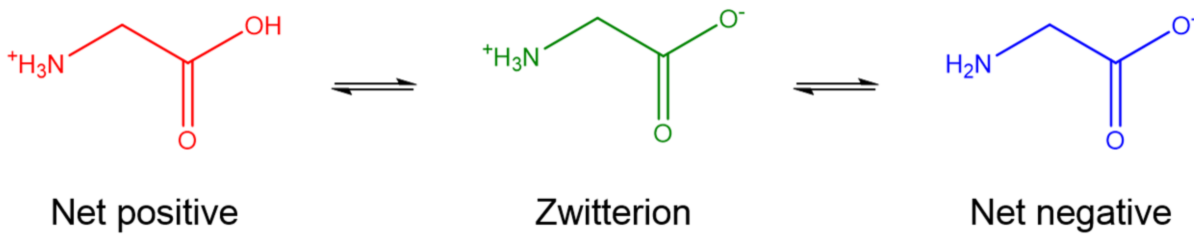

Figure S2. The chemical speciation of glycine as a function of solution pH: under acidic conditions, majority of glycine exhibits a net positive charge; under basic conditions, majority of glycine exhibits a net negative charge. At intermediate (near-neutral) pH conditions, glycine is charged but overall neutral. (Please, see Fig. S3 for further details)

Speciation curves of glycine at various pH.

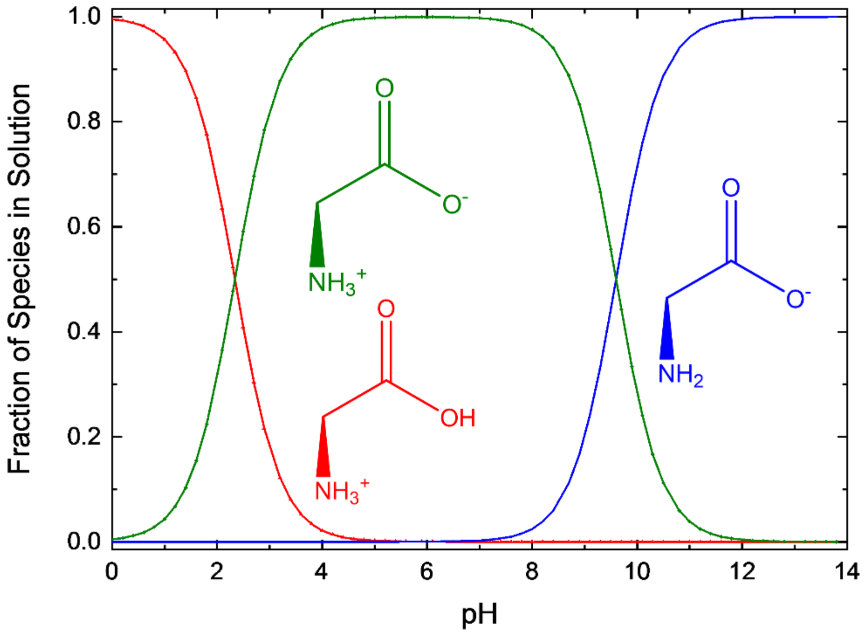

Figure S3. The fractions of different states of the amino acid glycine at different pH values.

References

1. Israelachvili, J. N., *Intermolecular and Surface Forces*. Third ed.; Academic Press, Elsevier Inc.: 2011.
